# Supplementary material for: Effect of poor glycemic control on the prevalence and determinants of anemia and chronic kidney disease among type 2 diabetes mellitus patients in Jordan: An observational cross-sectional study
Source: PLoS One. 2024 Nov 14;19(11):e0313627. doi: 10.1371/journal.pone.0313627 (PMC11563440; doi:10.1371/journal.pone.0313627)
Supplement: Attachment — Submitted filename: Response to Reviewers 2.docx [file pone.0313627.s001.docx]

Dear Editor,

We sincerely appreciate the opportunity to revise and resubmit our manuscript titled “Effect of Poor Glycemic Control on the Prevalence and Determinants of Anemia and Chronic Kidney Disease Among Type 2 Diabetes Mellitus Patients in Jordan: An Observational Cross-sectional Study" (Manuscript ID: [PONE-D-24-28044]). We are grateful for the constructive feedback provided by both you and the reviewers, which has substantially improved the quality of our manuscript.

We have carefully reviewed all the comments and have made the necessary revisions in response. Below, we provide a detailed point-by-point explanation of the changes made.

Thank you once again for your time and consideration.

Best regards,
Ahmed Al-Dwairi, PhD
___________________________________________________________________________________

1. Please remove any funding-related text from the manuscript.

Response: Thank you for your feedback. I have removed all funding-related text from the manuscript as per your request.

2. Your ethics statement should only appear in the Methods section of your manuscript. If your ethics statement is written in any section besides the Methods, please delete it from any other section.

Response: Thank you. I have ensured that the ethics statement is now only included in the Methods section and have removed it from any other sections of the manuscript.

3. Thank you for sharing the OSF repository link with us. However, users are required to request access to view the data. PLOS journals require authors to make all data necessary to replicate their study’s findings publicly available without restriction at the time of publication. We ask the authors to make the data publicly accessible and share the DOI with us (<https://help.osf.io/article/285-control-your-privacy-settings#Make-your-project-or-components-public>).

Response: Thank you for bringing this to my attention. I have adjusted the privacy settings on the OSF repository to ensure that the data is now publicly accessible without restriction.

Here is the updated DOI: 10.17605/OSF.IO/T3FYB
