# Supplementary material for: Effect of poor glycemic control on the prevalence and determinants of anemia and chronic kidney disease among type 2 diabetes mellitus patients in Jordan: An observational cross-sectional study
Source: PLoS One. 2024 Nov 14;19(11):e0313627. doi: 10.1371/journal.pone.0313627 (PMC11563440; doi:10.1371/journal.pone.0313627)
Supplement: Attachment — Submitted filename: Response to reviewers.docx [file pone.0313627.s002.docx]

Dear Editor,

We sincerely appreciate the opportunity to revise and resubmit our manuscript titled “Effect of Poor Glycemic Control on the Prevalence and Determinants of Anemia and Chronic Kidney Disease Among Type 2 Diabetes Mellitus Patients in Jordan: An Observational Cross-sectional Study" (Manuscript ID: [PONE-D-24-28044]). We are grateful for the constructive feedback provided by the reviewers. We have thoroughly addressed their comments and made the necessary revisions to enhance the manuscript.

To facilitate the review process, we have included a detailed point-by-point response that outlines how we have incorporated their suggestions into the revised manuscript.

Thank you for your consideration.

Sincerely,

Ahmed Al-Dwairi, PhD

__________________________________________________________________________

**Reviewer:** Methodology part needs to be rewritten with major focus on sample enrollment and sample size calculation. For analyte analysis, write down the method employed for determination of serum levels of insulin, fasting blood glucose, total cholesterol, and triglycerides.

**Response:** Thank you for your constructive feedback. In response to your comments, we have made the necessary revisions to the Methodology section, as outlined below:

*Sample Enrollment and Sample Size Calculation:*

We have now provided a detailed description of the sample enrollment process and sample size calculation. The sample size was calculated based on an estimated prevalence of type 2 diabetes mellitus (T2DM) of 15% in the adult population of Jordan, with a 95% confidence level and a 5% margin of error. The calculated sample size was 196 based on the standard formula for calculating sample size for population proportions:

n= Z2 × p × (1-p) /E2.

These details have been incorporated into the revised Methods section.

*Analyte Analysis:*

As per your request, we have clarified the methods employed for the determination of serum insulin, fasting blood glucose, total cholesterol, and triglycerides. The following updates were made:

- **Serum Insulin**: Serum insulin levels were measured using a validated enzyme-linked immunosorbent assay (ELISA), which provides high specificity and sensitivity.
- **Fasting Blood Glucose**: Glucose levels were analyzed using the glucose oxidase method, utilizing the cobas® modular analyzer system (Roche Diagnostics, Mannheim, Germany).
- **Total Cholesterol and Triglycerides**: Both total cholesterol and triglyceride levels were measured using enzymatic colorimetric assays, also performed on the cobas® modular analyzer system.

These methodological details have been added to the Methods section to enhance the clarity and accuracy of our analysis description.

We appreciate your insightful comments, which have significantly improved our manuscript. Thank you for your thorough review.

Sincerely,
Ahmed Al-Dwairi, PhD
